# Supplementary material for: CAPoxy: A feasibility study to investigate multispectral imaging in nailfold capillaroscopy
Source: PLoS One. 2026 Aug 12;21(8):e0329913. doi: 10.1371/journal.pone.0329913 (PMC13466010; doi:10.1371/journal.pone.0329913)
Supplement: Supplementary file 1 [file pone.0329913.s001.docx]

**Supplementary Information for:**

CAPoxy: a feasibility study to investigate multispectral imaging in nailfold capillaroscopy

Michaela Taylor-Williams^1,2,3^, Irsa Khalil^4^, Joanne Manning^5^, Graham Dinsdale^5^, Michael Berks^6^, Luca Porcu^2^, Sarah Wilkinson^4,5^, Sarah Bohndiek^1,2^* and Andrea Murray^4,5,7^*

1 Department of Physics, University of Cambridge, UK

2 Cancer Research UK Cambridge Institute, University of Cambridge, UK

3 Department of Biomedical Engineering, Johns Hopkins University, USA

4 Scleroderma and Raynaud’s Research Group, Division of Musculoskeletal and Dermatological Sciences, The University of Manchester, UK

5 Salford Care Organisation, Northern Care Alliance NHS Foundation Trust, UK

6 Division of Informatics, Imaging and Data Sciences, The University of Manchester, UK

7 National Institute for Health and Care Research (NIHR) Manchester Biomedical Research Centre (BRC) (Unique identifier: NIHR203308)

* Co-corresponding authors: Michaela Taylor-Williams email: [mtayl165@jhu.edu](mailto:mtayl165@jhu.edu). Sarah Bohndiek email: [seb53@cam.ac.uk](mailto:seb53@cam.ac.uk), tel: 01223 337267. Andrea Murray email:, tel:. 01612754292

**Contents of the supplementary file:**

- Supplementary Methods text:
  - Imaging system characterisation
  - Image processing and segmentation
  - Random forest classification
  - Statistical analysis
  - Visualisation of the ratio images
- Supplementary Figures 1-9
- Supplementary Tables 1-3

**Supplementary Methods**

*Imaging system characterisation*

The exposure time of the images (46.406 ms) was set using the uEye Cockpit Software (IDS Imaging) and the images taken were saved as portable network graphic (png) files. Images were acquired from an NBS 1963A resolution target (Thorlabs) for characterisation of pixel size and spatial resolution. The dimensions of the pixels composing the images were determined by measuring the pixel-to-distance ratio (1 px = 0.9379 µm) for the imaging system. This was calculated by finding the number of pixels across one element at ten different locations and averaging this distance, before dividing by the number of line pairs per millimetre for the element measured. The spatial resolution was also tested using the same NBS 1963A target and indicates visibility of 81 cycles/mm or 12 µm. This resolution is larger than the pixel-to-distance ratio, but this is not surprising since the 3 x 3 mosaic degrades the spatial resolution and the spatial resolution will also be modified by the imaging optics.

*Image processing and segmentation*

In order to understand systematic changes in the spectra, the images were analysed using Python. The image analysis process is illustrated in Supplementary Figure 3. First the capillary image $\left( I_{\mathrm{raw}} \right),$ the corresponding average white image ($I_{white}$), and average dark images ($I_{dark}$) were demosaicked into image arrays for each of the nine bands using linear interpolation. This process was self-coded in Python to enable flexibility in implementing these changes. These were then combined to correct the original capillary image ($I_{cor}$):

$$\begin{aligned} I_{cor}=\frac{I_{raw}-I_{dark}}{I_{white}-I_{dark}}\#Supp. Eqn. 1 \end{aligned}$$

Four different regions (R) of interest (Capillary, Edges_20_, Edges_40_ and Outside) were defined according to the following criteria:

Capillary: Each capillary that was visible was outlined in Labelbox, and the internal region of this segmentation forms the capillary mask, before exporting as a binary jpeg file. Often capillaries on the edges, or with incomplete shapes, were too faint or blurry to be defined easily and thus were not segmented. The consequence of not segmenting these capillaries is that they also contribute to the “outside” mask region. Also, when the two arms of the capillary could not easily be distinguished or were too small, the whole region was selected, including some image content that might be regarded as “not capillary.” To account for these imperfections, two masks indicating the surrounding area of the capillary were developed: Edges_20_ and Edges_40_.

Edges_20:_ In the case of the Edges_20_ mask, these were made using code to find the regions within 20 pixels of the capillary but not inside the capillary. Some of the edge region include areas of the capillary if it was not well defined.

Edges_40_: The Edges_40_ mask was similar, but the region within 40 pixels and not inside the capillary or Edges_40_ mask was excluded. This region is further away from the capillary, and it has the potential to overlap with other capillary regions if they are not outlined in the capillary mask.

Outside: The fourth mask of the “outside” region was calculated as all areas not included in the capillary, Edges_20_, and Edges_40_ masks. Some of the outside regions include parts of the nailfold that may have capillaries but were not in focus or defined, so they could not be outlined. Furthermore, it is not possible to subtract these regions easily, but it is presumed that the amount of capillary tissue is less than that in the capillary region.

The segmentation was performed on the demosaicked 553 nm image, where the contrast was highest for easiest visualisation. Masks (M) were manually created according to the above criteria.

$$\begin{aligned} M=\{M_{\mathrm{Capillary}}, M_{\mathrm{Edges}20}, M_{\mathrm{Edges}40}, M_{\mathrm{Outside}}\}\#Supp. Eqn. 2 \end{aligned}$$

$$\begin{aligned} R =\{I_{\text{cor}} \cdot M_{\text{Capillary}}, I_{\text{cor}} \cdot M_{\text{Edges20}}, I_{\text{cor}} \cdot M_{\text{Edges40}}, I_{\text{cor}} \cdot M_{\text{Outside}}\}\#Supp.Eqn. 3 \end{aligned}$$

$$\begin{aligned} \overline{R}\left( \lambda\right)=\frac{1}{n}\sum_{i=1}^{n} R_{i}\left( \lambda\right)\#Supp. Eqn. 4 \end{aligned}$$

The resulting images were then analysed to find the average ($\overline{R}$) in each region (with n pixels) for each band, such that four arrays with nine spectral points were made for each image. The four masks used are illustrated in Supp. Figure 4 on four example images.

Of the 382 images collected in the study, all were manually evaluated, but around half were not possible to segment for one or more of the following reasons: out of focus, no capillaries visible, motion artefacts, or poor image quality (Supp. Figure 4). As a result, image segmentation was performed on 186 images in total: 111 images from 10 different HC and 75 images from 7 different patients with SSc. Images from 2 of the HC and 3 of the patients with SSc were not used due to poor image quality or lack of visible capillaries; this is a rather normal occurrence in capillaroscopy imaging as not everyone’s capillaries are easily visible. The number of images assessed from each participant is stated in Supp. Table 3.

After masking, the spectra from the different regions were extracted for a spectral quality control inspection. Images one participant were discounted during this stage as the recorded spectra deviated substantially from the other spectra recorded (Supp. Figure 7).

To better understand the data and enable comparisons of recorded reflectance between participants, two different normalisations of the spectra collected were assessed:

Normalisation to the broadband filter intensity value ( $\overline{R_{\text{broadband}}}\left( \lambda\right)$):

$$\begin{aligned} \overline{R_{\text{broadband}}}\left( \lambda\right)=\frac{\overline{R}\left( \lambda\right)}{\overline{R}\left( \lambda=\text{broadband} \right)}\#Supp. Eqn. 5 \end{aligned}$$

Normalisation using a mean centering, the average of eight narrowband ($\overline{R_{meancentred}}$):

$$\begin{aligned} \overline{R_{meancentred}}\left( \lambda\right)=\frac{\overline{R}\left( \lambda\right)}{\sum_{i=narrowband} \overline{R}\left( \lambda\right)}\#Supp. Eqn. 6 \end{aligned}$$

The broadband filter or mean centre used was the average of the region of the image of interest, as this is most closely related to values of interest.

*Random Forest classification*

Random forest (RF) is an ensemble learning method used for classification that combines the outputs of multiple decisions trees to determine the optimum classification (4). The data were separated into a training and test sets with a 4:1 ratio of cross-validation. The RF was trained on the training data set, before being tested on the test data. This was done two different ways (Supplementary Figure 6):

- Overall classification: The data set was split only into HC and SSc regardless of the region of interest mask i.e. data from all regions was pooled and distinguished only according to disease status.
- Regional classification: The classifier was trained on each different spatial region, e.g., trained and then tested using HC and SSc capillary data. This was done four times over for each of the different regions.

The resulting classification metrics (accuracy, cross-validation (CV), precision, recall, and F1 score for each method were calculated to indicate the performance of each classification method (Supplementary Table 4).

*Statistical analysis*

The following sequential steps were used to develop and fit LMMs:

Step 1) Fit the mean model.

The mean model functional form was identified with (i) plots of overlaid observed and predicted mean model plots, (ii) residual plots, and (iii) numerical measures of relative model fit quality [Akaike information criterion (AIC), Bayesian information criterion (BIC) and likelihood ratio test (LRT)], all from models fit with Maximum Likelihood (ML) assuming independence.

We looked for a piecewise linear regression model (PLRM) over the eight spectral bands as reference statistical model (RSM). In the RSM the PLRM’s intercept was estimated at the lowest band (i.e. 553 nm) by disease status and region. The linear interaction between PLRM and disease status and the linear interaction between PLRM and region were introduced as predictors in the RSM.

Step 2) Determine variance and covariance structure.

Once the general form of the mean model was fixed, we evaluated the covariance structure between the repeated measurements taken on the same participant. Random effects of the intercept and slopes of the piecewise regression model, and the distribution of residual errors were assessed using graphical (i.e. histograms, Q-Q plots, scatter plots and semi-variograms), numerical summaries (i.e. mean and standard deviation) and numerical measures of relative model fit quality [AIC, BIC and LRT, all from models fit with restricted maximum likelihood (REML) estimation, the same fixed-effects structure, and the same choice of contrasts]. The random-effects structure of the RSM was defined by a random intercept for each participant, random slopes of the piecewise linear regression model for each participant and a nested random intercept for each image within each participant. Statistical independence between random intercepts and different random slopes of each participant was assumed in the RSM.

Homogeneity of residual variance was graphically and formally assessed. Because the assumption of homogeneity of residual variance was violated at different wavelengths, a variance function coefficient as RSMs’ parameter was used to describe the dependency of the residual variance on the wavelengths.

Step 3) Re-fit the statistical model.

Once the correlation structure was set, statistical models were re-fit with the chosen mean and covariance structures using REML.

Step 4) Hypothesis testing and estimation.

The primary and exploratory hypotheses tests were performed.

Sensitivity analyses were performed to identify pathological effects of outliers on conclusions of the RSM, and to develop and fit other reasonable statistical models whose conclusions could be contradictory respect to that of the RSM. After outliers have been identified, the RSM was fitted on the dataset without outliers. The analysis without outliers confirmed the results of the primary analysis. The following alternative statistical models were examined: α) the reference statistical model with other covariance structures of random effects and residuals; β) a more general piecewise regression model in which the interaction terms between disease status, region and piecewise linear regression terms over the eight spectral bands (Step 1) were added as predictors in the RSM. Different covariance structures of random effects and residuals were fitted, and outliers’ analysis was performed; γ) natural splines instead of PLRM over the eight spectral bands to develop and fit the RSM and the β model too. Different covariance structures of random effects and residuals were fitted, and outliers’ analysis was performed. Alternative data models confirmed the results of the primary analysis.

**Supplementary Figures**

**Supplementary Figure 1:** The (a) spectrum and (b) temporal stability of the white LED used in the system.

**Supplementary Figure 2:** (a) Image of group 29 on NBS 1963A Target that corresponds to 29 lines/mm. (b) Image of groups 72, 81, and 91; the delineation of the lines is visible in group 81; this corresponds with a spatial resolution of 12 µm resolution.

**Supplementary Figure 3:** The image processing begins with (a) raw images of the nailfold, white, and dark that are (b) demosaicked into nine band arrays. (c) They are then corrected using Equation 6.1. (d) These are then overlayed with the four masks (Equations 6.2 and 6.3) and the average in each of these regions is then found for each band to (e) create four spectra (Equation 6.4). (f) An example of a high-contrast image taken from the 553 nm band shows the four masks used.

**Supplementary Figure 4**: Example images with poor capillary visibility and artefacts. Some common problems with the images included: no capillaries visible or the capillaries were not fully visible, out of focus, or motion artefacts.

**Supplementary Figure 5:** Example masks applied to nailfold images: Example masks on four nailbed images from four particpants; two were HC, while the other two had SSc. The five horizontal images are as follows: the original image at 553 nm band with the image contrast increased, the capillary mask overlayed with the 553 nm band, the Edges_20_ mask overlayed with the 553 nm band, the Edges_20_ mask overlayed with the 553 nm band, and the outside mask overlayed with the 553 nm band.

**Supplementary Figure 6:** (a) The overall classification method combines the HC and SSc spectra into a combined dataset that is then split into testing and training. The training dataset is then used to train a RF classifier before testing on the test dataset to find the overall classification metrics. (b) The regional classification method combines the four different region HC and SSc spectra into four different region datasets, each of these is then split into testing and training. The regional training dataset is then used to train a RF classifier before testing on the regional test dataset to find the regional classification metrics. The regional classification metrics can then be averaged to assess the average performance of different classifiers.

Note: * The average spectra for each masked region (9 spectral points per region) are used as inputs for the Random Forest (RF) classifier. ** An 4:1 (80/20) training-to-test split was applied. *** Internal 5-fold cross-validation was performed on the training dataset to evaluate model robustness and minimize overfitting.The classification of the test dataset is compared with the disease state to find the accuracy, other metrics calculated are average CV, precision, recall, and F1-score.

**Supplementary Figure 7:** (a) The individual participant spectra for each of the mask regions and their 95% confidence interval (CI), measured using a Student’s t-test to account for sample size, are plotted. Participant in which there were no suitable images to analyse are not included as no spectra was measured. (b) The last four plots compare the average spectra of each participant according to their disease state to illustrate the variation.

**Supplementary Figure 8:** Example images illustrating the ratio of different regions for four different images: (a) Greyscale images of the 553 nm band and the corresponding colour bar, (b) raw ratio of 587 nm / 553 nm using the demosaiced images, (c) denoised ratio images using a 6 kernel median filter, (d) raw ratio of 629 nm / 553 nm using the demosaiced images, and (e) denoised ratio images using a 6 kernel median filter.

**Supplementary** **Figure 9:** The difference in reflectance between 80% and 100% oxygenated haemoglobin ( Reflectance[80% SO_2_] - Reflectance[100% SO_2_] ) with 15 nm bandwidth detection is shown. The centre wavelengths of 553 nm, 587 nm, and 629 nm are shown. A positive difference in reflectance indicates that 80% oxygenated haemoglobin is more reflective at that wavelength, while a negative reflectance indicates that 100% oxygenated haemoglobin is more reflective. Since this difference in reflectance is calculated for a 15 nm bandwidth window (assuming Gaussian shape), the center wavelength of the filter can be used to determine the expected effect. The difference in reflectance for the 553, 587, and 629 nm filters is -0.022, -0.011, and -0.024.

**Supplementary Table 1:** The classification accuracy, average CV, precision, recall, and F1-score for each of the models when trained on the whole dataset and on select ratios.

| **Metric** | **Description** | **Equation** |
| --- | --- | --- |
| Classification accuracy | The proportion of correct predictions by the model | $Accuracy=\frac{TP+TN}{TP+FP+TN+FN}$ |
| Average cross-validation (CV) | Average of the accuracy of different folds | $Average CV=\frac{\sum_{i=1}^{K=5} Accuracy of fold i}{K}$ |
| Precision | The rate of true positives out of predicted | $Precision=\frac{TP}{TP+FP}$ |
| Recall | The proportion of correct identification of the actual positives | $Recall=\frac{TP}{TP+FN}$ |
| F1 Score | Combines precision and recall in a harmonic mean to give a single score | $F1 Score=2 \times\frac{Precision \times Recall}{Precision+Recall}$ |

**Supplementary Table 2:** The classification accuracy and metrics when trained on the whole dataset, as shown in Supplementary Figure 6(a).

| Model | Accuracy | Average | CV | Precision Recall | F1-Score |
| --- | --- | --- | --- | --- | --- |
| RF | 0.84 | 0.73 | 0.84 | 0.83 | 0.83 |

**Supplementary Table 3:** Classification accuracy and metrics when using regional masks as shown in Supplementary Figure 6(b).

|  | Accuracies | | | | | Average Metrics | | | |
| --- | --- | --- | --- | --- | --- | --- | --- | --- | --- |
| Model | Average | Capillary | Edges20 | Edges40 | Outside | CV | Precision | Recall | F1-Score |
| RF | 0.82 | 0.87 | 0.82 | 0.82 | 0.79 | 0.71 | 0.83 | 0.82 | 0.82 |
